# Recurrent aseptic meningitis in adults: a potential indicator of undetermined autoinflammatory disease

**DOI:** 10.3389/fimmu.2026.1842256

**Published:** 2026-05-11

**Authors:** Martin Martinot, Samuel Ardois, Elodie Chauvet, David Cavestro, Philippe Blanche, Thomas Papo, Anne Murarasu

**Affiliations:** 1 Service de Maladies Infectieuse, Hôpitaux Civils de Colmar, Colmar, France; 2 Service de Médecine Interne, CHRU Rennes, Rennes, France; 3 Service de Médecine Interne, CHRU Montpellier, Montpellier, France; 4 Département de Médecine Interne, Hôpital Cochin, APHP, Paris, France; 5 Service de Médecine Interne, Hôpital Bichat, Université Paris Cité, Paris, France; 6 Service de Médecine Interne, CHRU Tours, Tours, France

**Keywords:** aseptic meningitis, autoinflammation, autoinflammatory diseases, recurrent meningitis, USAID

## Abstract

**Background:**

Autoinflammatory diseases (AIDs) are characterized by recurrent episodes of inflammation caused by primary immune system dysregulation. Central nervous manifestations, including aseptic meningitis (AM), are rare in AIDs. In this study, we identified six patients with undetermined AIDs characterized by recurrent aseptic meningitis (RAM).

**Methods:**

Patients referred to Centre National de Référence des Maladies Auto-Inflammatoires for AM with an undetermined diagnosis were included. AIDs with RAM were defined by two or more episodes of aseptic meningitis (>10 leukocytes) with a predominance of neutrophils (>50%) and an association with autoinflammatory elements (i.e., fever, C-reactive protein [CRP] elevation) and the absence of an alternative diagnosis.

**Results:**

Six patients (four men, two women) were included, all of whom presented with fever and elevated CRP levels (≥30 mg/L), and five patients had clinical meningitis syndrome. The median patient age at disease onset was 41 years (range, 28–49). The frequency of AM was 1–24 episodes/year, and the duration of episodes was ≤8 days. Associated clinical symptoms included cutaneous manifestations (rash [n = 3], Raynaud’s syndrome [n = 1]), arthromyalgia (n = 2), and central neurological or ophthalmologic manifestations (n = 2). All patients underwent genetic analysis focusing on AIDs, and variants of undetermined significance within four autoinflammatory genes were noted in three patients. All patients benefited from classical therapeutic regimens of AIDs including corticosteroids, colchicine, and anti-interleukin (IL)-1 receptor or anti-IL6 antibodies.

**Conclusion:**

RAM was identified as a potential indicator for undetermined AIDs. Physicians should be aware of this condition and provide adequate analysis and treatment.

## Introduction

Recurrent aseptic meningitis (RAM) is characterized by repeated episodes of meningitis unrelated to the infectious process and the normalization of cerebrospinal fluid (CSF) levels between successive consecutive events. Aseptic meningitis (AM) can develop under various circumstances (1, 2), especially in systemic diseases, drug-induced meningitis, and carcinomatous meningitis. Autoinflammatory diseases (AIDs) are clinical disorders caused by defects in or dysregulation of the innate immune system characterized by recurrent or persistent inflammation and the absence of a primary pathogenic role for the adaptive immune system (3). However, there is increasing evidence that AIDs involve a link between innate and adaptative immunity (4). AIDs can be associated with monogenic or polygenic diseases, and they encompass a wide range of manifestations, some of which affect the nervous central system but rarely cause AM (5, 6). Undetermined AIDs (UAIDs) are not related to the previously described AIDs (7), and difficult to diagnose. In our experience, some patients developed RAM as the central and sometimes exclusive manifestation of possible UAIDs, accompanied by recurrent fever and inflammatory syndrome. Such patients did not have phenotypically or genotypically determined AIDs, but they responded well to treatments administered for AIDs.

In this study, we describe six cases of USAIDs registered at Le Centre National de Référence des Maladies Auto-Inflammatoires et de l’Amylose Inflammatoire (CeRéMAIA) in which RAM was the main clinical manifestation.

## Materials and methods

Patients with RAM who were referred to CeRéMAIA between 2017 and 2025 without a definite diagnosis were included in the study. RAM was defined as two or more episodes of AM with a predominance of neutrophils (>50%), normalization of clinical symptoms between episodes, and the presence of autoinflammatory elements, such as fever and elevated C-reactive protein (CRP) levels. Differential diagnoses excluded by the evaluating physician based on patients’ medical history and biological investigations to establish the diagnosis of RAM included the following: infectious diseases (negative microbiological samples including direct CSF examination, negative blood and CSF cultures, negative bacterial culture or negative bacterial or fungal antigen test, negative viral/bacterial or fungal polymerase chain reaction assay including HSV1-2, or negative HIV serology); systemic diseases such as Behçet’s disease, sarcoidosis, lupus erythematosus, Sjögren’s disease, or Vogt–Koyagie–Harada disease through testing including antinuclear antibody, antineutrophil cytoplasmic antibody, glycosylated ferritin, rheumatoid factor, or human leukocyte antigen test; drug hypersensitivity; neoplastic or paraneoplastic meningitis (negative antineuronal antibodies and CSF analysis); and other well-defined monogenic or polygenic AIDs. Brain magnetic resonance imaging was also performed to eliminate characteristic cerebral diseases such as abscess and tumor.

## Results

Eleven patients with AM as the main manifestation were evaluated. Five patients were excluded because informed consent could not be obtained.

### Sociodemographic characteristics and medical history

All six evaluated patients (four men, two women) presented with RAM, fever, and elevated CRP levels (mean, 68.8 mg/L) and neutrophils counts (≥50%) in CSF, and five of six patients exhibited typical clinical meningitis syndrome (one patient had febrile headache without meningeal stiffness). In five patients, CSF parameters were controlled within normal levels between flares. The mean age at onset was 41 years (range, 28–49). The incidence of AM ranged 1–24 episodes/year, and the duration of episodes was ≤8 days except for one case (60 days). The associated clinical symptoms included cutaneous manifestations (rash, n = 3; Raynaud’s syndrome, n = 1), arthromyalgia (n = 2), and central neurological or ophthalmologic manifestations (n = 2; **Table 1**).

**Table 1:** Demographic and clinical manifestations of patients with recurrent aseptic meningitis genotypes and corresponding treatments.

Sex, age at onset (years)	Duration of follow-up from the initial episode (years)	Duration of episode (days), frequency per year	Associated symptoms			Brain MRI	Main genetic results (Sanger, NGS, WES)	Treatments
			Neurological and ophthalmologic features	Cutaneous and rheumatologic features	Abdominal aches			
M, 43	19	8, 1–2/year	0	Maculopapular rash	0	Nonspecific signals T2 FLAIR hyperintensity	MEVF neg TNRF neg NLRP3 neg WES neg UBA1 (VEXAS) neg IRAK4 neg	COL (+)
F, 37	8	5, 1/month	Interstitial keratitis	Maculopapular rash, arthromyalgia	0	Normal	MEVF neg TNRF neg NLRP 3 vC2113C>A MKV neg	COL (+) CS (+) Anti-IL1R (−) Anti-IL-6 (tocilizumab) (+)
F 46	34	<8, 2–4/year	Myelitis with hemiparesia and a neurological bladder	Maculopapular rash	0	Right occipital periventricular lesion with a moderately increased diffusion-weighted imaging signal	MEFV vV726+/−, TNFRS1A vR92Q +/− NLRP3 neg MKV neg	COL (−) CS (+) MMF, AZA (−) Anti-IL-1R (−) Anti-IL-6 (tocilizumab) (+)
M, 28	15	<8, 1–2/year	0	0	0	Normal	MEFV neg TNFR neg NLR12 vA297P23 (frameshift and the appearance of a stop codon) MKV neg	COL (−) CS (+) Anti-IL-1R (canakinumab) (+)
M, 49	12	60, 1–2/year	0	Arthromyalgia	0	Nonspecific signals on T2-weighted imaging	MEVF neg TNRF neg NLRP3 neg UBA1 (VEXAS) neg	COL (−) CS, 4 mg/day, increased during flares (+)
M, 44	4	4, 2/month	0	Raynaud’s syndrome Lichen	0	Normal	MEVF neg TNRF neg NLRP3 neg MKV neg WES neg UBA1 (VEXAS) neg IRAK4 neg	COL (−) HXQ (−) Anti-IL-6 (tocilizumab) (+)

NGS, next-generation sequencing; WES, whole-genome sequencing; COL, colchicine; CS, corticosteroids; MTX, methotrexate; anti-IL-1R, anti-interleukin-1 receptor antibody; anti-IL-6, anti-interleukin-6 antibody; MMF, mycophenolate mofetil; AZA, azathioprine; CYC, cyclophosphamide; HXQ, hydroxychloroquine; MRI, magnetic resonance imaging. Neg no pathologic mutation detected (+): positive response; (−): negative response.

### Laboratory results at baseline

Episodes were associated with elevated CRP levels (mean, 68.8 mg/L; range, 35–120), elevated CSF leukocyte counts (mean, 1378 leukocytes/mm^3^; range, 108–4260; 85.5% neutrophils; range, 73–92), elevated CSF protein levels (mean, 1.12 g/L; range, 0.6–1.56), and CSF glucose glycorrhachia (mean, 2.82 mmol/L; range, 2–3.25).

### Genetic analysis

All patients underwent genetic analysis focusing on AIDs, and variants of undetermined significance were detected within four autoinflammatory genes in three patients (**Table 1**).

### Treatments and outcomes

All patients were treated with colchicine, with one patient receiving colchicine monotherapy, and four patients received bolus steroids during flares (continuously and exclusively in one patient each). Three patients were treated with anti-interleukin (IL)-1 receptors (successfully in one case), and three patients received anti-IL-6 therapy with favorable outcomes. Other (unsuccessful) treatments included mycophenolate mofetil, azathioprine, cyclophosphamide, and hydroxychloroquine (**Table 1**).

## Discussion

In this study, we reported a case series of six patients with RAM as the central manifestation of potential UAIDs AIDs includes both well characterized monogenic diseases but also in large proportion of patients (>70%) unidentified pathogenic variant or established diagnosis based on defined criteria (8, 9). These forms are named UAIDs and their diagnosis is based on repeated documentation of biologic inflammatory syndrome over 6 months and exclusions of other symptoms as this was the case for our 6 patients. In adults, the most common manifestations are recurrent fever, fatigue, myalgia, arthralgia, cutaneomucous and digestive features, ENT or ocular involvement (8, 9). We propose to include RAM as the possible manifestations of UAIDs. However, our cohort, comprising a very small number of patients is only a hypothesis-generating observation warranting prospective study.

Symptom onset occurred in adulthood in all cases (mean age of onset, 41 years), and patients experienced between 1 and 24 attacks per year. However, some patients experienced headaches without requiring hospitalization. In addition, some attacks were not evaluated. Some patients presented with other symptoms, mainly cutaneous, rheumatologic, neurologic, or ophthalmologic symptoms, which are commonly associated with UAIDs (10, 11), Although not definitive for a diagnosis of AIDs, neurological symptoms, including AM, are uncommon in AIDs and UAIDs. However, these symptoms have been described previously in other conditions, such as cryopyrin-associated periodic syndromes, familial Mediterranean fever, tumor necrosis factor receptor-associated periodic syndrome, complement factor I deficiency, VEXAS syndrome, and phosphatidylinositol glycan anchor biosynthesis class T mutation (11–15).

Though no mutation allowing for a certain diagnosis of classic AIDs has been observed, genetic variants of undetermined significance within four autoinflammatory genes were observed in three patients, as previously described (16). Variants of undetermined significance (VUS) are, by definition, of uncertain pathogenic relevance, and their use as supporting evidence for an autoinflammatory etiology needs therefore to be considered with cautious. The occurrence of variants in the causative genes of monogenic AIDs can promote polygenic disease, although an unidentified monogenic disease is also possible. Because of retrospective design of our analysis, whole-exome sequencing or next-generation sequencing using a recently introduced panel including included novel genes of interest such as VEXAS and IRAK4 was performed in only two patients. This absence of whole-exome sequencing related to the retrospective design of the study limits the ability to exclude monogenic disease, which is a prerequisite for the UAID classification (9). Thus, prospective analysis are needed to further define the genes potentially involved in RAM more precisely (17). However, such prospective studies are difficult to perform because of the rarity of RAM. In the absence of criteria for AIDs response to treatment targeting innate immunity such as colchicine or cytokine inhibitors (IL-1, IL-6, TNF, JAK) may confirm the diagnosis (8). All our patients with UAIDs have been treated with various treatment regimens, including corticosteroids, colchicine, and anti-inflammatory drugs, and cytokine inhibitors. Only one patient out of 3 responded to anti-IL1 a key treatment of AIDs and tocilizumab was the most effective agent, underscoring the possible role of IL-6 pathway in the UAID with RAM pathophysiology.

Regarding study limitations, the possibility of bias could not be excluded because of the retrospective design of the study, which used data collected several years after the first episode of AM. Furthermore, comprehensive data collection including mild symptoms potentially evocative of AIDs was difficult because of the retrospective study design, the length of the study period, and the treatments administered, which influenced the pattern of symptoms and the number of episodes. Although physicians eliminated most differential diagnoses, including Mollarets meningitis based on a lack of Mollarets cells (18), high CRP levels, a lack of CSF lymphocytosis, and repeated negative HSV PCR including multiplex panel (19), a workup framework was not a prerequisite for the diagnosis of RAM. However, in our opinion, long-term patient follow-up without the emergence of an alternative diagnosis in conjunction with the treatments performed represent strong criteria in favor of UAIDs and the exclusion of other causes.

## Conclusion

In conclusion, we identified RAM as a potential indicator of UAIDs. The diagnosis of this condition relies on physician awareness; therefore, the discovery of RAM should encourage physicians to engage in AID-oriented evaluations and AI treatments. Failure to diagnose AIDs can have life-threatening consequences. However, further studies are needed to confirm our proposal and better define this entity and its treatments.

## Data Availability

The raw data supporting the conclusions of this article will be made available by the authors, without undue reservation.
